# Wilson disease in Northern Portugal: a long-term follow-up study

**DOI:** 10.1186/s13023-022-02245-5

**Published:** 2022-02-23

**Authors:** Isabel Garrido, Margarida Marques, Rodrigo Liberal, Hélder Cardoso, Susana Lopes, Guilherme Macedo

**Affiliations:** 1 Gastroenterology and Hepatology Department, Centro Hospitalar Universitário de São João, Alameda Prof. Hernâni Monteiro, 4200-319 Porto, Portugal; 2 World Gastroenterology Organization (WGO) Porto Training Center, Porto, Portugal

## Abstract

**Introduction:**

Wilson disease is an autosomal recessive disease of liver copper metabolism with predominant hepatic and neurological manifestations. Long-term data on the clinical follow-up and treatment efficacy are limited due to the low frequency of the disease. We evaluated a large cohort of Wilson disease patients from Northern Portugal during a 20-year follow-up period.

**Methods:**

Twenty-four patients, diagnosed from 1975 to 2020 in a tertiary care center in Portugal, were retrospectively evaluated according to their clinical presentation, therapies and outcomes.

**Results:**

Most of the patients were males (54%), with a median age at diagnosis of 19 years old (interquartile range 15–25). The main manifestations of Wilson disease were hepatic (71%) and neurological (25%). Family history was positive in 5 (21%) patients. Four patients (17%) presented with acute liver failure and fifteen (63%) individuals had cirrhosis at diagnosis. Penicillamine therapy was used by 11 (46%) patients, while trientine and zinc were given to 8 (33%) and 1 (4%) patient, respectively. Ten (42%) individuals underwent liver transplantation. The majority of patients (83%) had stable disease or improved outcomes during follow-up.

**Conclusion:**

This is the largest cohort of adult patients with Wilson disease reported in Northern Portugal. We show that Wilson disease has favorable outcomes with long overall survival, assuming adherence to therapy and lack of other insults to their liver.

## Introduction

Wilson disease is a rare inherited disorder of copper accumulation. Defective biliary excretion of cooper leads to its accumulation in the body, particularly in the liver and brain [1]. The clinical manifestation, which includes different forms of liver disease, hemolytic anemia and neuropsychiatric disorders, can occur at any age and at different steps in the course of the disease [2]. Differential diagnosis may be challenging as its different phenotypic features can assume variable presentation forms, such as acute hepatitis (similar to any other acute cause of hepatitis), chronic hepatitis and cirrhosis (as routine histologic changes are nonspecific); the neurologic symptoms may be overlocked as primary psychologic or psychiatric disorders. Unless appropriate lifelong treatment is started, it will naturally progress to chronic failure and death. In fact, failure in diagnosis is the most common cause of death in these patients [3]. The importance of early recognition of clinical manifestations is reflected in the timely initiation of therapy, leading to a better prognosis [4–6].

The overall prevalence of Wilson disease is estimated at 1 in 30.000 in non-isolated populations [7]. Given this, coupled with Portugal´s low population density, clinicians often have difficulty acquiring adequate exposure to this disease. The aim of this study was to assess the evolution of liver disease and possible long-term complications in patients with Wilson disease followed in an adult Liver Center.

## Methods

We have retrospectively assessed the clinical evolution of all patients diagnosed with Wilson disease and followed at a tertiary hospital in Porto, Portugal in the last 25 years. The diagnosis was based on clinical signs, biochemical (ceruloplasmin levels < 20 mg/dL, urinary cooper > 100 µg/24 h) and histologic findings (liver copper levels > 250 µg/g dry weight), according to Leipzig score [8, 9]. The presence of Kayser–Fleischer rings was explored through a slit lamp examination. Liver biopsy histology slides were evaluated by a specialized liver histopathologist. Liver copper concentration was determined through atomic absorption spectroscopy. Collected data were enrolled in an SPSSS program, including the demographic data, clinical features, laboratory workup, treatment modalities and outcomes.

## Results

A total of 24 patients were diagnosed with Wilson disease between 1975 and 2020 in our Center. The physicians primarily involved in the diagnosis were gastroenterologists/hepatologists (67%), pediatricians (17%), neurologists (8%), ophthalmologists (4%) and internal medicine specialists (4%). The median age at diagnosis was 19 years old (interquartile range 15–25), in 13 male and 11 female patients. All of them were index cases and family history was able to track 5 other patients (followed in other institutions).

In Table 1, the demographic and clinical aspects are described. We have considered the presenting features as hepatic when patients presented abnormal liver function tests and/or signs of clinical decompensation as jaundice (25%), ascites (17%) or acute liver failure (17%); neurologic when tremor, dystonia or dysarthria was dominant (25%). One patient was referred by an ophthalmologist after observing Kayser–Fleischer rings in a routine observation for myopia.

**Table 1:** Characteristics of Wilson disease patients

	Sex	Age at diagnosis (years old)	Familiar history	Predominant features	Neurologic symptoms	Kayser–Fleischer rings	Staging of liver fibrosis (Metavir score)	Hepatic liver failure	Treatment	Liver transplant	Outcome	Follow-up (years)
1	Male	18	No	Hepatic	No	No	F4	No	Penicillamine	No	Alive	29
2	Male	22	No	Neuropsychiatric	Yes	Yes	F3	No	Penicillamine >> Trientin	No	Alive	4
3	Male	43	No	Hepatic	No	No	F4	No	Trientin	No	Alive	4
4	Female	15	No	Hepatic	No	No	F1	No	Zinc	No	Alive	25
5	Female	12	No	Hepatic	No	No	F2	No	Penicillamine	No	Alive	8
6	Male	30	No	Hepatic	No	Yes	F4	No	Penicillamine	Yes	Alive	20
7	Female	8	No	Hepatic	No	No	F2	No	Trientin	No	Alive	14
8	Female	13	Yes	Neuropsychiatric	Yes	Yes	F4	Yes	–	Yes	Alive	15
9	Female	16	No	Hepatic	No	No	F4	No	–	Yes	Alive	29
10	Male	22	Yes	Hepatic	No	Yes	F4	No	–	Yes	Alive	14
11	Male	18	No	Ophthalmologic	No	Yes	F1	No	Penicillamine	No	Alive	32
12	Male	25	No	Neuropsychiatric	Yes	Yes	F4	No	–	Yes	Alive	20
13	Female	25	No	Neuropsychiatric	Yes	Yes	F4	No	Penicillamine	No	Death	12
14	Male	8	No	Hepatic	No	No	F1	No	Trientin	No	Alive	18
15	Male	18	Yes	Hepatic	No	No	F4	No	Penicillamine >> Trientin	No	Alive	17
16	Male	9	Yes	Hepatic	No	Yes	F4	No	Penicillamine	No	Alive	18
17	Male	22	No	Hepatic	No	Yes	F4	Yes	–	Yes	Alive	22
18	Female	50	No	Hepatic	No	No	F2	No	Penicillamine >> Trientin	No	Alive	15
19	Female	18	No	Hepatic	No	Yes	F4	Yes	–	Yes	Alive	22
20	Male	25	No	Neuropsychiatric	Yes	Yes	F4	No	–	Yes	Death	7
21	Female	16	No	Neuropsychiatric	Yes	Yes	F4	No	Penicillamine	Yes	Alive	19
22	Female	19	No	Hepatic	Yes	Yes	F4	Yes	–	Yes	Alive	16
23	Male	21	Yes	Hepatic	Yes	No	F2	No	Trientin	No	Alive	20
24	Female	32	No	Hepatic	No	No	F2	No	Penicillamine >> Trientin	No	Alive	20

We have obtained liver histology in all patients, in 10 (42%) via transplant approach due to severe coagulation impairment. Four patients presented an acute liver failure with histology having cirrhosis and reticular collagen, justifying liver transplant indication. Hemolytic anemia was present in only three of these acute patients. Significantly, 15 (63%) individuals had already cirrhosis and in 5 (21%) steatohepatitis features were predominant.

Kayser–Fleischer rings were objectively observed in 13 (54%) patients but in 100% of predominant neurologic features; in those with predominant liver affection, Kayser–Fleischer rings were present in only 6 (35%) patients.

Twenty-four hours urinary cooper was reported to be over 500 µg in all patients as serum ceruloplasmin was also below 15 mg/dL. Liver copper was above 300 µg/g dry weight in 17 (71%) individuals.

Genetic analysis was performed in 8 (33%) patients, 5 of whom had genetic mutations. Seven distinct mutations were detected in at least one of the alleles. The c.3402delC at exon 15 was the most common mutation, with an allelic frequency of 30%, followed by the c.3207C>A substitution at exon 14, with an allelic frequency of 20%

Treatment modalities included trientine (900–1500 mg/day) in 8 (33%) patients, penicillamine (750–1500 mg/day) in 7 (29%) and zinc therapy (150 mg/day) in 1 (4%) patient. The first therapeutic approach was penicillamine in 11 patients but changing to trientine was needed in 4 due to toxicity (overall penicillamine toxicities rate of 4/11, 36%)—hematologic in 2 severe cases (hemolytic anemia and aplastic anemia) and dermatologic in the other 2 (alopecia and lichen planus). No adverse effects were reported with trientine or zinc treatment.

Patients were followed for a period of 18 years (interquartile range 14–22). They were evaluated in gastroenterology and/or neurology consultation every 1–3 months during the initial phase of treatment and for those experiencing worsening of symptoms or side effects of medications, and every 3–6 months thereafter. Liver function tests, serum copper and ceruloplasmin were evaluated twice a year and urinary copper at least yearly [9]. For patients on chelation therapy, blood count and urine analysis were also monitored biannually. Adherence to treatment was evaluated during follow-up, as well as side effects. For those with cirrhosis, screening and surveillance of esophageal varices were performed according to the European Association for the Study of the Liver guidelines [10]. In addition, these individuals were screened twice a year for hepatocellular carcinoma.

During follow-up, the treatment adherence rate was 92%. The majority of patients (83%) had stable or improving liver function tests. Two individuals (8%) had ascites, requiring diuretic therapy, and six individuals (25%) presented gastro-esophageal varices, one of them with variceal hemorrhage. There were no cases of hepatocarcinoma. One patient (4%) experienced documented episodes of neurological decline and five individuals (21%) developed mental health problems despite the stable liver disease.

A liver transplant was performed in 10 (42%) patients and one other individual refused to be included in the liver transplant list and died with liver failure 12 years after diagnosis. One of the transplanted patients died with posttransplant lymphoproliferative disorder 5 years after the orthotopic transplant. The overall survival rate of those transplanted patients was 90%. One of our transplanted patients, with a tacrolimus basal regime, accomplished an uneventful pregnancy, 14 years ago.

Liver histology in the acute liver failure presenting patients was decisive in confirming significant cooper accumulation in cirrhotic livers and clarified the diagnosis (as in 1 of these patients there was a presumptive diagnosis of acute alcoholic hepatitis, autoimmune hepatitis in 2 and leptospirosis in one young teenager). The transplant was accomplished in 1–3 months in the acute liver failure cases and 9–18 months in cirrhotic candidates.

The overall survival rate of this cohort of patients was 92%, reflecting a careful follow-up and good adherence to therapy (and immunosuppressors, if needed).

## Discussion

This study is the first description of a long-term follow-up of Wilson disease patients living in Northern Portugal. Our study draws on the experience of 24 patients, demonstrating the variable presentation, treatment history and course of Wilson disease.

The median age at diagnosis was similar to that previously described by other authors [11]. There were, however, some individuals with onset of symptoms on the extremes of age. In fact, two of our patients were over 40 years of age at diagnosis, providing further support that late-onset Wilson disease may not occur as rarely as previously believed. Thus, Wilson disease must be considered at all ages. With regard to gender, there was an equal distribution of the disease.

Although we have not evaluated the time lag from the appearance of the first symptoms to the diagnosis, some authors have shown a significantly long period of time to establish the diagnosis of Wilson disease [12, 13]. The delay in diagnosis is devastating because the longer the disease progresses without treatment, the more likely it is that liver and/or brain damage will be permanent. The difficulties in diagnosing Wilson disease are related to the different clinical presentations of the disease.

The most important clinical manifestations of copper overload among our patients were hepatic (71%) and neuropsychiatric (25%). Our results show no differences in the age of the onset of the symptoms among patients with predominant hepatic versus neuropsychiatric disease (18 vs. 20 years old, *p* = 0.973). All of our patients with neurological predominant manifestations had documented Kayser–Fleischer rings. On the other hand, the presence of Kayser–Fleischer rings was substantially lower among those with a hepatic presentation, reinforcing the idea that this feature is not a sensitive test in the diagnosis of non-neurological Wilson disease.

It is important to screen first-degree relatives of individuals with Wilson disease for early detection and treatment, which helps in a better long-term outcome. Specific testing for known mutations or haplotype analysis should be the method used [9]. In our cohort, 5 (21%) patients had a positive family history, leading to early referral.

Currently, there is no universally accepted treatment for Wilson disease. Nevertheless, once the diagnosis is made, lifelong therapy is required. We show the clinical course of the disease over a period of 20 years under treatment. The majority of our patients were treated with trientine or penicillamine and are on the same medication they started with. Nevertheless, 36% of patients on penicillamine changed treatment due to the side effects of therapy. In fact, the traditional use of penicillamine as the mainstay of Wilson disease treatment is increasingly challenged and may no longer be true.

It is well known that lifelong treatment compliance is often difficult for Wilson disease patients. Failure to comply with lifelong therapy leads to recurrent symptoms and liver failure [9]. During follow-up, the majority of our patients had a stable or improved outcome, highlighting not only the importance of early diagnosis but also compliance to therapy. In fact, long-term treatment of patients with penicillamine and/or trientine is effective in resolving clinical and biochemical markers of the disease and improving prognosis. On the other hand, the results of medical therapy for patients who presented with hepatic liver failure (17%) were disappointing as all patients needed liver transplantation. Pos-transplant survival rate was 100%, reflecting the importance of early referral to a transplant center in these cases.

There were no reports of hepatocellular carcinoma in our cohort. In fact, some authors have already suggested that copper and its receptors have a protective role against hepatic carcinogenesis [14, 15]. This may be the reason for the extreme rarity of hepatocellular carcinoma in patients with Wilson's disease and possibly in other liver diseases with hepatic copper overload.

There are a few data on fertility and the outcome of pregnancy in Wilson disease. In our cohort, only one patient became pregnant after liver transplantation, having a successful pregnancy and healthy term birth. Indeed, several successful pregnancies and uneventful full-term deliveries have been described in mothers with Wilson disease undergoing treatment [16, 17]. On the other hand, pregnancy does not seem to have an adverse effect on the clinical course of the disease.

Similarly, data on quality of life and social features are scarce in the literature. Since the disease has a long overall survival with adequate treatment, these features need to be analyzed. Some authors suggested that Wilson disease has a high risk for depressive disorders [18–20]. Therefore, physicians must be alert and maintain an active assessment for depression.

## Conclusion

Our retrospective study presents a cohort of adult patients with Wilson disease who are managing their disease quite well, assuming adherence to therapy and lack of other insults to their liver. To our knowledge, this is the largest cohort of patients with Wilson disease reported in Northern Portugal.

## Data Availability

Data sharing not applicable to this article as no datasets were generated or analysed during the current study.
